# Estimation of Salivary 8-Hydroxydeoxyguanosine (8-OHdG) as a Potential Biomarker in Assessing Progression towards Malignancy: A Case-Control Study

**DOI:** 10.31557/APJCP.2020.21.8.2325

**Published:** 2020-08

**Authors:** Arulmozhi Nandakumar, Priyadharsini Nataraj, Amritha James, Rajkumar Krishnan, Mahesh K M

**Affiliations:** 1 *Clinical Consultant, India. *; 2 *Department of Oral Pathology and Microbiology, SRM Dental College, University Chennai, Ramapuram, India. *; 3 *Department of Ophthalmology, Panimalar Medical College and Hospital, Chennai, India. *

**Keywords:** Oral submucous fibrosis, oral squamous cell carcinoma, 8-hydroxydeoxyguanosine, oxidative stress

## Abstract

**Objective:**

Squamous Cell Carcinoma is almost always preceded by potentially malignant disorders in the oral cavity before malignant transformation. Characterization of 8-OHdG from the saliva offers a relatively non-invasive, simple and efficient methodology for monitoring oxidative stress in subjects of Premalignant oral disorders (PMOD) and Oral Squamous Cell Carcinoma (OSCC). Hence the aim of the current study is to estimate the levels of salivary 8-hydroxydeoxyguanosine (8-OHdG) as a potential DNA Damage Biomarker in OSMF and OSCC patients in comparison to healthy individuals to assess disease progression from potentially malignant oral disorder to frank malignancy.

**Materials and Methods:**

The study was conducted among 90 patients [Oral Squamous cell carcinoma (n=30) and Oral Submucous Fibrosis (n=30) and healthy gender and age matched controls (n=30)]. 4ml of unstimulated saliva was collected from each of the subjects and was subjected to Sandwich ELISA for the quantification of salivary 8-OHdG. Statistical analysis was done using ANOVA, and p value was set at ≤0.05.

**Results:**

The mean age of OSCC patients were 56.8±11.8 years. Smoking was the most prevalent adverse habit among this group (66.6%) followed by Smokeless tobacco chewers (40%). The mean age of OSMF patients was 46.2± 9.8 years. Smokeless tobacco was the most predominant habit among the OSMF patients (83.33%) followed by smoking (33.33%). The mean OHdG levels among the controls was 6.59±1.47 (ng/dl) and almost doubled in patients of OSMF 13.89±1.96(ng/dL) and further raised in OSCC patients 19.96 ± 2.11 (ng/dL). These levels showed a highly significant difference (p <0.0001) in mean on comparison by using one-way ANOVA. Pearson correlation between the groups were also statistically significant (p=0.000).

**Conclusion:**

There were significant differences in the concentration of salivary 8-OHdG between healthy controls, OSMF, and OSCC patients. Hence, 8-OHdG can be used as a novel biomarker of DNA damage to assess disease progression.

## Introduction

‘Oxidative stress’ is employed to describe the relation between free radicals and disease and can be defined as the state at which oxidation of the cell exceeds the antioxidant repair systems in the body (Rai et al., 2014; Katakwar et al., 2016). Reactive Oxygen Species (free radicals) formed due to oxidative stress play a vital role in the etiology and evolution of major degenerative disorders including oral cancer and precancerous conditions (Bahar et al., 2007). The highly reactive and unstable ROS produce oxidative harm which ultimately leads to deoxyribonucleic acid (DNA) base modiﬁcations and single as well as double-strand breakages (Gupta et al., 2014). ROS and oxidative stress actively influence all the three stages of carcinogenesis and through varied events and pathways, interact with and damage cellular components, and contribute to neoplastic transformation in these cells (Ma et al., 2006; Kruk et al., 2019).

The hydroxyl radical (HO•) is one of the most critical oxygen-free radical causing damage to fundamental biomolecules like membrane lipids, cellular proteins and DNA. To start with, the HO• reaction leads to the production of free-radical induced DNA adducts, and later by a chain of reactions and electron abstraction, 8-hydroxy-2-deoxyguanosine (8-OH-dG) is generated (Valavanidis et al., 2009). The correlation of the levels of oxidative DNA damage with the mutation frequencies and cancer incidence depends on the availability of sensitive methods for the analysis of slight levels of DNA damage. Since the 8-OHdG quantiﬁcation at low levels of DNA damage is possible it is the most reliable biomarker for the oxidative DNA damage (Henderson et al., 2010; Sanchez et al., 2018). 

Squamous Cell Carcinoma is almost always preceded by pre-malignant lesions in the oral cavity prior to malignant transformation but there is no steadfast objective indicator for the behavior of these lesions or their imminent transformation (Fernández-Olavarría et al., 2016). 

Characterization of 8-OHdG from the saliva offers a relatively non-invasive, simple and efficient methodology for monitoring oxidative stress in subjects of Premalignant oral disorders (PMOD) and Oral Squamous Cell Carcinoma (OSCC). Very few studies have compared the quantitative progression of 8-OHdG levels through PMODs and Cancer. 

Hence the current study aims to Estimate the levels of salivary 8-hydroxydeoxyguanosine (8-OHdG) as a potential DNA Damage Biomarker in OSMF and OSCC patients in comparison to healthy individuals to assess disease progression from potentially malignant oral disorder to frank malignancy.

## Materials and Methods

The study was conducted in the out-patient department of SRM Dental College after obtaining approval from the Institutional Review Board. 

*Inclusion and exclusion criteria*

The study population included newly diagnosed and histopathologically proven cases of Oral Squamous cell carcinoma (n=30) and Oral Submucous Fibrosis (n=30) with healthy gender and age matched controls (n=30) prior to commencement of treatment. Patients and controls with co-morbidities and other systemic disorders were excluded from the study.

*Saliva Collection*

After obtaining informed and written consent, the complete history was recorded and an intra-oral examination was done. Approximately 4ml of unstimulated saliva was collected from each of the subjects in quiet, resting conditions. The saliva was then transferred to sterile centrifuge tubes and centrifuged at 3,000 rpm at 4 degree centigrade for 20 minutes. The resulting supernatant was separated in 1ml aliquots and stored in a -80C freezer prior to laboratory estimation. 

*ELISA*

Sandwich ELISA was done for saliva samples obtained, for quantification of salivary 8-OHdG, using Biotin double antibody sandwich technology to assay the Human 8-Hydroxy-desoxyguanosine (8-OHdG) from ELISA kit by BioAssay Technology Laboratory.

*Statistical Analysis*

Statistical analysis was done using SPSS software version 16 for windows; SPSS Chicago, IL, USA. Following the derivation of the OD value and subsequent calculation of the quantity of 8-OHdG in the saliva samples, the resultant values were tabulated and expressed as a mean value. To compare the mean 8-OHdG values among the three groups, one-way analysis of variance test was done using GraphPad PRISM 5 software (CA, USA) and the values were considered significant when p values were 0.05 or less. Correlation coefficient analysis was used to compare the habit metrics and 8-OHdG levels. 

## Results

The mean age of OSCC patients were 56.8±11.8 years; ranging from 39-70 years. Smoking was the most prevalent adverse habit among this group (66.6%) followed by Smokeless tobacco chewers (40%) and 4 patients showed combined habits and 2 patients had no history of habits. The mean age of OSMF patients was 46.2± 9.8 years ranging from 31-60 years. Smokeless tobacco was the most predominant habit among the OSMF patients (83.33%) followed by smoking (33.33%) and 5 patients had a combined habit. Alcohol consumption was prevalent exclusively in the male population 60% (of males) in the OSMF and 62% (of males) in the OSCC group. The mean age of the control group was 52.4±8.4 years and they presented with no adverse habits. The demographic details of the patients are tabulated in Table 1.

The mean OHdG levels among the controls was 6.59±1.47 (ng/dl) and almost doubled in patients of OSMF 13.89±1.96(ng/dL) and further raised in OSCC patients 19.96 ± 2.11 (ng/dL). These levels showed a highly significant difference (p <0.0001) in mean on comparison by using one-way ANOVA (Table 2).

**Figure 1:**
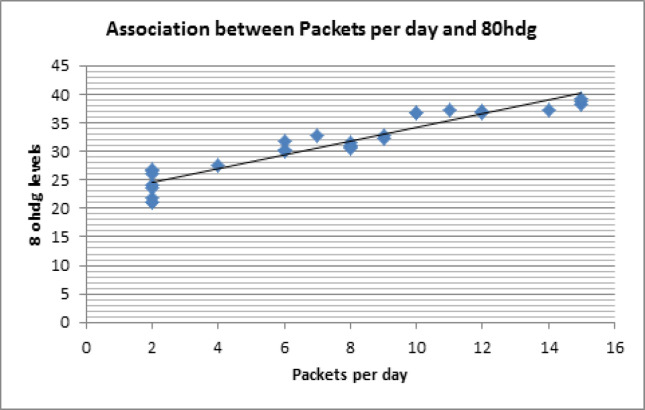
Correlation between Packet Years and 8- OHdg Levels

Among OSCC patients who were smokers, total pack years were calculated by multiplying the number of cigarettes per day and duration of smoking in years (frequency duration). There was a highly positive correlation between the increase in the number of pack years in OSCC patients and the increase in 8-OhDG levels (r=0.923) (Figure 1). This correlation was statistically significant(p=0.000).

**Figure 2:**
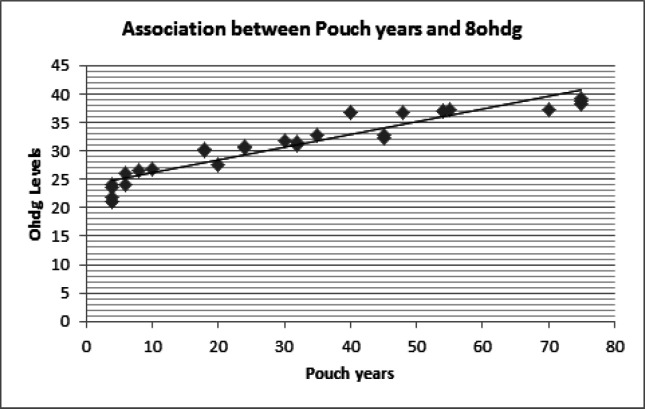
Correlation between Pouch Years and 8- OHdg Levels

Among the OSMF patients since tobacco chewing was the most prevalent habit, a modification of pack years for smoking was adapted and total pouch years was calculated. The pouch year is a product of the number of packets chewed per day and duration of chewing in years. There was a highly positive correlation between the increase in the number of pouch years in OSMF patients and the increase in 8-OhDG levels (r=0.910). This correlation was statistically significant (p=0.000) (Figure 2).

No significant correlation was obtained between alcohol consumption and 8-OHdG levels among the OSMF or OSCC groups (p=0.335). 

**Table 1:** Demographic Data of the Subjects among the Three Groups

	Age	Gender		Habits				
	(mean± SD)	M	F	Smoker	Chewer	Combined habit	Alcoholic	None
				n (%)	n (%)	n (%)	n %)	n (%)
OSMF	46.2±9.8	20	10	10 (33.3)	25 (83.3)	5 (16.6)	12 (40)	-
OSCC	56.8±11.8	24	6	20 (66.6)	12 (40)	4 (13.3)	15 (50)	2 (6.7)
Control	52.4±8.4	20	10	-	-	-	-	30 (100)

**Table 2:** Comparison of Salivary 8-OHdG Value (ng/dL)Using One Way ANOVA

Groups	N	Mean ± SD	*P*-value
OSMF	30	13.89±1.96	<0.0001
OSCC	30	19.96±2.11	<0.0001
Controls	30	6.59±1.47	

## Discussion

Oxidative stress is an established model of pathogenesis in premalignant oral disorders (PMODs). A previous study by Vlokova et al., (2012), involved the determination of markers of oxidative stress in leukoplakia, erythroplakia and lichen planus and they concluded that deficiency of anti-oxidant status and increase in lipo-peroxidation was established etiology in PMODs. In the current study, we analyzed the levels of 8-OHdG in the saliva of Oral submucous fibrosis and Oral Squamous cell carcinoma patients to understand the behavior of this biomarker through the progression of the disease from a healthy normal individual through premalignant conditions and frank malignancy. 

The role of oxidative stress in the development model of OSMF has been recently characterized by decreased antioxidant activity and increased serum malondialdehyde levels (Shakunthala et al., 2015). The malignant transformation rate of oral submucous fibrosis (OSF) has been reported to be around 7.6% over a 17-year period and it has been reported in several epidemiological studies mainly in the Southern states of India (Trivedy et al., 2002). Nevertheless, there are no potential markers to understand the malignant transformation of this disorder.

Increased oxidative stress in OSCC has been previously studied by Kumar et al., (2012) and they found a significant increase in 8-OHdG levels in saliva of OSCC as opposed to controls. They concluded that the profound redox imbalance in the subjects was responsible for the development of OSCC. 

Dietary nitrates also contribute to oxidative stress in the oral cavity. Oxidative stress also increases with age and in the presence of other chronic degenerative diseases (Zhang et al. 1999; Takahama et al., 2009). Hence age and gender matched controls were chosen for comparison and the control population was devoid of any chronic diseases. 

Smoking was the commonest adverse habit in OSCC due to carcinogenic nitrosamines in smoke (Hecht, 2014). Pre-malignant condition patients had an increasing trend of chewing smokeless tobacco. Pandya et al., (2009) in their study showed that commercial smokeless products such as paan masala, gutkha, and mawa have higher concentrations of areca nut and appear to cause OSMF more rapidly than self-prepared conventional quid, which contains smaller amounts of areca nut. 

The mean 8-OHdG values in saliva were progressively increased from controls to OSMF and OSCC patients. This gradient increase in 8-OHdG reflects the increase in DNA damage and oxidative stress environment in these conditions. Totan et al., (2015) in their study evaluated disease-related biomarkers in saliva and serum of OLP patients. They detected significant differences between the OLP and control groups in serum and saliva for 8-OHdG. Kumar et al., (2012) reported an increase in 8-OHdG levels in OSCC patients compared to controls and predicted it as an excellent marker for oxidant stress in carcinoma.

There was a high correlation between the increase in smoking habit metrics and salivary levels of 8-OHdG in OSCC patients. This is similar to previous studies in urinary excretion of 8- OHdG among lung cancer subjects that concluded that it was higher in current smokers compared to controls (Loft et al., 2006). Another study compared the 8-OHdG levels among smokers, non-smokers and former smokers and they deduced that maximum 8-OHdG levels were present in current smokers and those who smoked greater than 10 cigarettes per day (Lodovici et al., 2000).

The OSMF patients showed an increasing trend of paan chewing and a significant positive correlation was achieved between the habit metrics of paan and 8-OHdG levels in these patients. Jacob et al., (2004) showed a clear dose dependent relationship for both frequency and duration of chewing areca without tobacco in the development of OSMF. In the study by Shreshtha et al., (2012), they demonstrated decreased serum antioxidant levels in paan chewers with habit metrics of increased duration of chewing and increased packets per day. They concluded that chewing pan with tobacco, caused oxidative stress in the oral cavity and the chewers were in imminent danger of cellular damage. Thus the increase in OSMF incidence and habit metrics could be attributed to the accumulation of free-radicals from tobacco alkaloids and increased oxidative stress as indicated by the 8-OHdG values. 

In the current study, no significant correlation was obtained between alcohol consumption and 8-OHdG levels among either OSMF or OSCC groups. However, in another study, alcohol consumption increased oxidative stress in cancer patients. They concluded that chronic alcoholism leads to increased oxidative stress and low antioxidant levels and combined habit of smoking and alcohol consumption further aggravated the disease process (Nagamma et al., 2012).

Our study also has certain limitations, we were not able to expand our study to include various age groups because of the prevalence of systemic disorders in those subjects which would give rise to erroneous overestimation of 8-OHdG levels. Measurement of 8-OHdG levels has certain limitations by itself. This biomarker is highly sensitive but not specific. Accumulation of 8-OHdG is associated with a variety of disorders including cancers and neurodegenerative disorders. Also precise and reproducible quantification of 8-OHdG levels varies with different techniques. This study was also restricted to the urban population, and hence further studies are needed include the rural population as well. This study can be further expanded by correlating 8-OHdG levels with clinical staging and histopathological grading of oral squamous cell carcinoma and other potentially malignant disorders.

In conclusion, the present study elicits the fact that the mean salivary 8-OHdG levels showed significant differences not only between the cases and controls but also between patients with OSMF and OSCC, with OSCC showing the highest mean 8-OHdG levels. Thus salivary 8-OHdG can be used as a novel biomarker of DNA damage to assess disease progression from OSMF to OSCC.

## Conflict of interest

No conflict of interest.
